# Trajectory of physical activity frequency and cancer risk: Findings from a population-based cohort study

**DOI:** 10.1186/s11556-023-00316-5

**Published:** 2023-03-09

**Authors:** Thi Phuong Thao Tran, Ngoc Minh Luu, Thi Tra Bui, Minji Han, Min Kyung Lim, Jin-Kyoung Oh

**Affiliations:** 1 Department of Cancer Control and Population Health, National Cancer Center Graduate School of Cancer Science and Policy, 323 Ilsan-ro, Ilsandong-gu, Goyang-si, Gyeonggi-do 410-769 Republic of Korea; 2 Hanoi University of Public Health, Hanoi, Vietnam; 3 Hanoi Medical University, 1 Ton That Tung Street, Dong Da district, Hanoi, Vietnam; 4 Division of Cancer Prevention, National Cancer Center, 323 Ilsan-ro, Ilsandong-gu, Goyang-si, Gyeonggi-do 410-769 Republic of Korea; 5 Department of Social and Preventive Medicine, College of Medicine, Inha University, 100 Inha-ro, Michuhol-gu, Incheon, 22212 Republic of Korea; 6 National Cancer Center, Graduate School of Cancer Science and Policy, Goyang-si, 323 Ilsan-ro, Ilsandong-gu, Goyang-si, Gyeonggi-do 410-769 Republic of Korea

**Keywords:** Physical activity, Cancer incidence, Group-based trajectory modeling, South Korea

## Abstract

**Background:**

Physical activity (PA) changes throughout an individual’s life, but the association between such changes and cancer risk seems to be overlooked in the literature. Thus, this study aimed to examine the association between the trajectories of PA frequency and cancer incidence among middle-aged Korean adults.

**Methods:**

A total of 1,476,335 eligible participants (992,151 men and 484,184 women) aged ≥40 years from the National Health Insurance Service cohort (2002–2018) were included. Assessment of PA frequency was a self-reported measure, based on the question: “How many times per week do you perform exercise that makes you sweat?”. PA frequency trajectories (i.e., trajectory classes of change in PA frequency) from 2002 to 2008 were identified using group-based trajectory modeling. Cox proportional hazards regression was used to assess the associations between the PA trajectories and cancer incidence.

**Results:**

Five PA frequency trajectories over 7 years were identified: persistently low (men:73.5%; women:74.7%), persistently moderate (men:16.2%; women:14.6%), high-to-low (men:3.9%; women:3.7%), low-to-high (men:3.5%; women:3.8%), and persistently high (men:2.9%; women:3.3%). Compared with persistently low frequency, maintaining a high PA frequency was associated with a lower risk of all cancers (Hazard ratio (HR) = 0.92, 95%CI = 0.87–0.98) and breast cancer (HR = 0.82, 95%CI = 0.70–0.96) among women. There was a lower risk for thyroid cancer among men in the high-to-low (HR = 0.83, 95%CI = 0.71–0.98), low-to-high (HR = 0.80, 95%CI = 0.67–0.96), and high PA trajectories (HR = 0.82, 95%CI = 0.68–0.99). There was a significant association between moderate trajectory and lung cancer in men (HR = 0.88, 95%CI = 0.80–0.95), in both smoking and non-smoking men.

**Conclusion:**

Long-term persistent high frequency of PA as part of the daily routine should be widely promoted and encouraged to reduce the risk for all cancer development in women.

**Supplementary Information:**

The online version contains supplementary material available at 10.1186/s11556-023-00316-5.

## Background

The protective effect of physical activity (PA) on cancer risk via multiple potential mechanisms, such as reduction in circulating estrogen levels, insulin resistance, and inflammation, has been well-documented [1]. Strong evidence has shown that PA has a protective effect and reduces the risk for colon, breast, and endometrial cancer [1], while the impact of PA on the decreased risk for esophageal, lung, and liver cancer was suggestive [2]. Additionally, PA was reported to reduce weight gain, and this was indirectly attributed to a lower risk for obesity-related cancers [1]. However, such scientific evidence was accumulated from observational studies that investigated the association between PA at a single time point (i.e., baseline) and cancer outcomes. In fact, our behaviors, pertaining to performing PA, continuously change throughout the life course; this could modify the effects of PA on cancer risk suggested in existing evidence.

Recently, group-based trajectory modeling (GBTM) was developed as a novel approach that overcomes the disadvantages of the traditional method. GBTM can fully capture behaviors accounted for within-individual variation throughout the life course, and it has been commonly utilized for determining risky behaviors, such as tobacco use and alcohol consumption. Although GBTM has been increasingly applied to identify PA trajectories in relation to mortality and several disease outcomes [3–6], its association with cancer seems to be typically overlooked.

The association between the trajectory of PA and cancer risk has not been well-explored. To date, only one case-control study has investigated the impact of PA trajectory on pancreatic cancer risk [7]. The trajectory of moderate and vigorous PA from the 20s to 50s age was identified, including six latent groups: persistent inactivity, low activity, increasingly active, high activity with substantial decrease, high activity with a slight decrease, and persistent high activity. The results showed that none of these trajectories was significantly associated with the risk for pancreatic cancer. Thus, further investigation with a stronger study design, such as cohort study, is needed to elucidate the causal association between the trajectory of PA and cancer risk.

In South Korea, a high proportion of adults participate in insufficient PA [8], and it had been observed to increase from 24.6% in 2008 to 42.9% in 2014 [9]. The transition of PA status could affect cancer incidence; however, no studies have been dedicated to this issue in South Korea. Thus, we aimed to examine the association between different trajectories of PA frequency and cancer incidence among middle-aged Korean adults.

## Methods

### Study population

This study used data from a nationwide population-based cohort study that used the database provided by the National Health Insurance Services (NHIS) in South Korea [10]. In brief, the NHIS is a mandatory single-payer insurance provider that conducts a non-payment general health examination program for all insured adults biennially. The participation rate of this program was 74.1% in 2019 [11]. The data of 5,544,985 enrollees aged ≥40 years who underwent health examination in 2002–2003 were used. After excluding individuals with missing information regarding sex, age, and PA, 1,476,335 cancer-free individuals (992,151 men and 484,184 women) had information on PA frequency four times from the cohort between 2002 and 2008, were included and followed-up until 2018. As this study used anonymous secondary data, the study was exempted from review by the Institutional Review Board of the National Cancer Center, Korea (NCC2018–0279). This study was conducted according to the Declaration of Helsinki.

### PA trajectory

The frequency of PA was measured using a questionnaire as part of the general health examination, the main question used was: “How many times per week do you perform exercise that makes you sweat?”, and the five responses were 1) none, 2) 1–2 times, 3)3–4 times, 4)5–6 times, or 5) almost every day. We decided to determine the trajectories of PA frequency from 2002 to 2008 because the questionnaire has changed since 2009. As the general health examination was recommended biennially, four 2-year period time points (2002–2003, 2004–2005, 2006–2007, and 2008) were used to measure the trajectories of PA frequency. As aforementioned, we aimed to determine PA trajectories during the longest observable duration; therefore, we chose the exposure period of 7 years, from starting of the database (i.e., 2002) to the end of time before revising the health examination questionnaire (i.e., 2008).

**Fig. 1:**
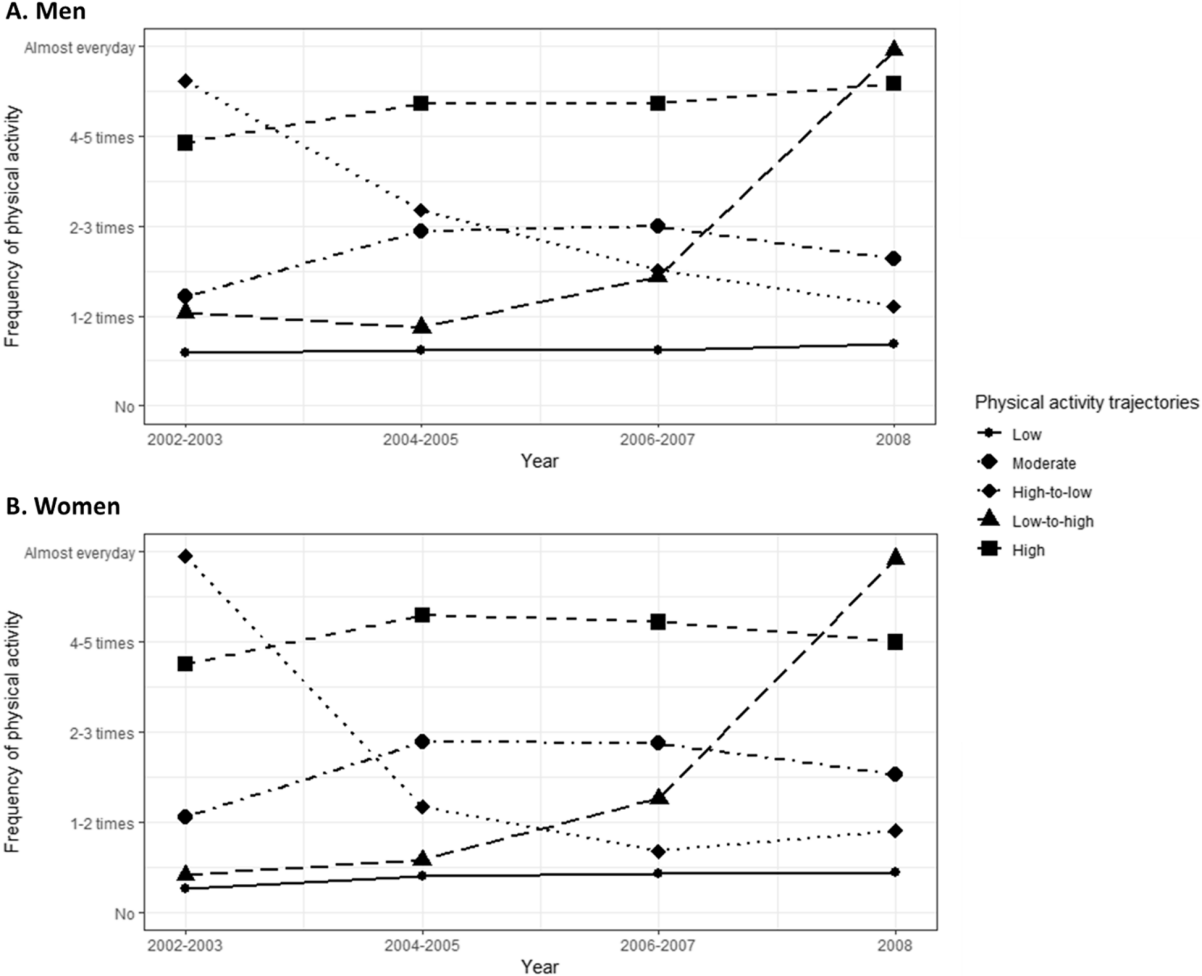
Trajectories of physical activity frequency over 7 years. **A**) Men, **B**) Women

PA trajectories were identified using the GBTM method proposed by Nagin with the PROC TRAJ in SAS [12]. The maximum number of trajectories was chosen based on the findings of a systematic review, in which the number of PA trajectories throughout the life-course commonly ranged from 3 to 5 [13]. Additionally, the maximum number of trajectories recommended is six; therefore, we tested on a maximum number of 6 trajectories. The process of choosing polynomial components was performed as the general rule, following the tutorial by Andruff [14]. Therein, initial testing involved a model with two cubic components (syntax ‘ORDER 3 3’). Once the only one component showed significant results, a model with one cubic component and one quadratic component (syntax “ORDER 3 2” or “ORDER 2 3) was tested. If significance were shown in none of the cubic component, the model’s quadratic components would be assessed (syntax “ORDER 2 2). Then, a model with one quadratic and one linear component (syntax “ORDER 1 2” or “ORDER 2 1”) or one with two linear components (syntax “ORDER 1 1”) was evaluated if the model’s quadratic components for two trajectories were not significant. Eventually, if all polynomial components of a model were significant, the analysis for two trajectories was finished, and the Bayesian information criterion (BIC) values and proportion of group membership (i.e., the percentage of each trajectory) were noted. Testing in three, four, five and six trajectories was repeated through this process, separately for men and women, until the best-fitting models was found. As recommended, the best-fitting model was chosen based on the smallest Bayesian Information Criteria (BIC) value, and the proportion of each group membership was ≥5% [15]. In our study, although we decreased the number of groups within the model, one trajectory group remained to have a low proportion (around 3.0% of the study population). Thus, five trajectories of PA frequency were identified in both men and women: low, moderate, high-to-low, low-to-high, and high (Fig. 1). Supplemental Table 1 summarizes the best-fitting models based on the number of groups. For the final models, the average posterior probabilities for the group 1 to 5 in men were 0.93, 0,88, 0,81, 0.95, and 0.92, respectively, and those in women were 0.81, 0.96, 0.94, 0.93, and 0.91, respectively, all of which were higher than the recommended cut-off value of 0.7 [16].

### Cancer outcome

The International Classification of Diseases 10th edition (ICD-10) codes were used to evaluate the incidence of all cancer types (C00–C97) and several specific cancers, including colon and rectal (C18–20), liver (C22), lung (C33, C34), breast (C50), corpus uteri (C54), and thyroid gland cancers (C73). Furthermore, a special code for cancer claims (V193) was additionally used to identify cancer occurrence during the follow-up period. All participants were followed-up until the date of cancer onset, death, or the end of the follow-up period (December 31, 2018).

### Covariates

Covariates were retrieved from baseline (2002–2003). Sex and age were included, and income levels were divided into quartiles, from Q1 (lowest income) to Q4 (highest income).

Behavioral risk factors were measured, including smoking status, body mass index (BMI), and alcohol consumption. Smoking status was categorized into three groups: non-smoker, former smoker, and current smoker. BMI was classified as underweight (< 18.5 kg/m^2^), normal (18.5–22.9 kg/m^2^), overweight (23–24.9 kg/m^2^), and obesity (≥25 kg/m^2^), according to the World Health Organization (WHO) obesity standard for the Asian population [17]. Alcohol consumption was classified into the following groups: rarely drinking, 2–3 times/month, 1–2 times/week, 3–4 times/week, and almost every day. Additionally, the Charlson Comorbidity Index (CCI) was also calculated for inpatients using ICD-10 codes [18].

### Statistical analysis

Descriptive statistics were used to describe the variables of participants’ characteristics. PA trajectories were identified based on the GBTM approach using PROC TRAJ in SAS 9.4 software. Cox proportional hazards regression was used to estimate the risk for cancer. In the multivariate model, we adjusted for age, income level, smoking status, alcohol consumption, BMI, and CCI score. We additionally adjusted for chronic viral hepatitis (i.e., B18 in ICD-10) in the multivariate model for liver cancer. For sensitivity analysis, we examined the association between PA trajectories and cancer incidence by smoking status and BMI groups. All statistical analyses were stratified by sex and performed using SAS 9.4 (SAS Institute, Inc., Cary, NC, USA).

## Results

More than two-thirds of the study participants had a low frequency of PA (73.51% in men; 74.66% in women), while approximately 15% and only 3% had a moderate and high frequency of PA during approximately 7 years, respectively.

The baseline characteristics according to the trajectories PA frequency in men and women are shown in Table 1 and Table 2, respectively. Compared to individuals with low PA frequency, both men and women with a higher frequency of PA tended to be richer and overweight/obese. Men in the low PA frequency category tended to be younger, had a higher proportion of current smokers, and were less likely to consume alcohol every day.

**Table 1:** Baseline characteristics according to trajectories of physical activity frequency in men

	Total men (*n =* 992,151)		Low (*n =* 729,353; 73.51%)		Moderate (*n =* 160,349; 16.16%)		High-to-low (*n =* 38,917;3.92%)		Low-to-high (*n =* 34,570;3.48%)		High (*n =* 28,962;2.92%)	
	n	%	n	%	n	%	n	%	n	%	n	%
**Age, mean (SD)**	48.22 (7.48)		47.86 (7.27)		48.08 (7.12)		50.17 (8.55)		51.74 (8.82)		51.37 (8.93)	
**Income group (quartile)**												
Missing	29,129	2.94	19,347	2.65	6425	4.01	1339	3.44	1141	3.30	877	3.03
Q1	80,894	8.15	61,258	8.40	10,628	6.63	3387	8.70	3679	10.64	1942	6.71
Q2	128,898	12.99	101,356	13.9	14,605	9.11	5051	12.98	5023	14.53	2863	9.89
Q3	249,408	25.14	192,940	26.45	33,379	20.82	9070	23.31	8174	23.64	5845	20.18
Q4	503,822	50.78	354,452	48.60	95,312	59.44	20,070	51.57	16,553	47.88	17,435	60.2
**Frequency of alcohol drinking**												
Missing	2411	0.24	1705	0.23	398	0.25	83	0.21	130	0.38	95	0.33
Rarely drinking	303,543	30.59	223,528	30.65	46,408	28.94	12,232	31.43	12,241	35.41	9134	31.54
2–3 times/month	233,790	23.56	172,750	23.69	39,108	24.39	8323	21.39	7232	20.92	6377	22.02
1–2 times/week	300,236	30.26	220,014	30.17	51,587	32.17	11,238	28.88	9177	26.55	8220	28.38
3–4 times/week	108,266	10.91	78,876	10.81	17,824	11.12	4564	11.73	3665	10.60	3337	11.52
Almost everyday	43,905	4.43	32,480	4.45	5024	3.13	2477	6.36	2125	6.15	1799	6.21
**Smoking status**												
Missing	3975	0.40	2800	0.38	656	0.41	137	0.35	212	0.61	170	0.59
Never smoker	387,765	39.08	275,028	37.71	66,394	41.41	17,033	43.77	15,576	45.06	13,734	47.42
Former smoker	181,538	18.30	124,731	17.10	35,768	22.31	8228	21.14	6078	17.58	6733	23.25
Current smoker	418,873	42.22	326,794	44.81	57,531	35.88	13,519	34.74	12,704	36.75	8325	28.74
**BMI group**												
Missing	363	0.04	263	0.04	67	0.04	13	0.03	15	0.04	5	0.02
Underweight	15,784	1.59	13,489	1.85	1233	0.77	402	1.03	452	1.31	208	0.72
Normal	324,246	32.68	251,733	34.51	42,951	26.79	11,501	29.55	10,276	29.73	7785	26.88
Overweight	292,618	29.49	212,012	29.07	49,704	31.00	11,798	30.32	10,049	29.07	9055	31.27
Obesity	359,140	36.20	251,856	34.53	66,394	41.41	15,203	39.07	13,778	39.86	11,909	41.12
**Charlson Comorbidity Index**												
0	978,354	98.61	719,590	98.66	158,072	98.58	38,285	98.38	33,907	98.08	28,500	98.40
≥ 1	13,797	1.39	9763	1.34	2277	1.42	632	1.62	663	1.92	462	1.60

**Table 2:** Baseline characteristics according to trajectories of physical activity frequency in women

	Total women (*n =* 484,184)		Low (*n =* 361,492; 74.66%)		Moderate (*n =* 70,560; 14.57%)		High-to-low (*n =* 17,757; 3.67%)		Low-to-high (*n =* 18,582; 3.84%)		High (*n =* 15,793; 3.26%)	
	n	%	n	%	n	%	n	%	n	%	n	%
**Age, mean (SD)**	49.91 (8.40)		49.66 (8.46)		49.26 (7.56)		53.07 (9.09)		52.27 (8.37)		52.16 (8.33)	
**Income group (Quartile)**												
Missing	3497	0.72	2452	0.68	616	0.87	136	0.77	155	0.83	138	0.87
Q1	117,370	24.24	93,806	25.95	12,356	17.51	4108	23.13	4712	25.36	2388	15.12
Q2	89,622	18.51	70,641	19.54	9772	13.85	3336	18.79	3512	18.90	2361	14.95
Q3	93,175	19.24	69,464	19.22	12,766	18.09	3909	22.01	3869	20.82	3167	20.05
Q4	180,520	37.28	125,129	34.61	35,050	49.67	6268	35.30	6334	34.09	7739	49.00
**Frequency of alcohol drinking**												
Missing	4700	0.97	3234	0.89	843	1.19	187	1.05	219	1.18	217	1.37
Rarely drinking	387,548	80.04	290,200	80.28	55,418	78.54	14,163	79.76	15,359	82.66	12,408	78.57
2–3 times/ month	59,371	12.26	44,201	12.23	9416	13.34	1986	11.18	1835	9.88	1933	12.24
1–2 times/week	25,814	5.33	18,980	5.25	3929	5.57	1055	5.94	902	4.85	948	6.00
3–4 times/week	3942	0.81	2890	0.80	552	0.78	184	1.04	147	0.79	169	1.07
Almost everyday	2809	0.58	1987	0.55	402	0.57	182	1.02	120	0.65	118	0.75
**Smoking status**												
Missing	**8652**	1.79	6086	1.68	1521	2.16	303	1.71	370	1.99	372	2.36
Never smoker	464,317	95.90	346,912	95.97	67,537	95.72	17,058	96.06	17,761	95.58	15,049	95.29
Former smoker	4439	0.92	3365	0.93	679	0.96	102	0.57	165	0.89	128	0.81
Current smoker	6776	1.40	5129	1.42	823	1.17	294	1.66	286	1.54	244	1.54
**BMI group**												
Missing	**318**	0.07	254	0.07	43	0.06	9	0.05	8	0.04	4	0.03
Underweight	10,910	2.25	9123	2.52	1076	1.52	259	1.46	272	1.46	180	1.14
Normal	202,280	41.78	155,071	42.90	28,897	40.95	6265	35.28	6294	33.87	5753	36.43
Overweight	125,801	25.98	91,923	25.43	19,442	27.55	4852	27.32	5056	27.21	4528	28.67
Obesity	144,875	29.92	105,121	29.08	21,102	29.91	6372	35.88	6952	37.41	5328	33.74
**Charlson Comorbidity Index**												
0	478,216	98.77	357,178	98.81	69,696	98.78	17,475	98.41	18,282	98.39	15,585	98.68
≥ 1	5968	1.23	4314	1.19	864	1.22	282	1.59	300	1.61	208	1.32

During the 9,368,662 person-years of follow-up, 84,703 men developed cancer. In the age-adjusted model, compared to the low category, men with a moderate trajectory of PA had a lower risk for all cancers (HR = 0.97, 95%CI = 0.95–0.98), and specific cancer of colorectum (HR = 0.92, 95%CI = 0.88–0.97) and lung (HR = 0.76, 95%CI = 0.72–0.81). A significantly lower risk for lung cancer was also observed in men with high-to-low and high frequency trajectory of PA. After adjusting for other potential confounders, only significant effect of the moderate trajectory of PA on a lower risk for lung cancer incidence remained (HR = 0.88, 95%CI = 0.82–0.93). Additionally, compared to the low trajectory, there was a lower risk for thyroid cancer among men in the high-to-low (HR = 0.83, 95%CI = 0.71–0.98), low-to-high (HR = 0.80, 95%CI = 0.67–0.96), and high trajectories (HR = 0.82, 95%CI = 0.68–0.99) (Table 3).

**Table 3:** HRs and 95% CIs for the association between physical activity trajectory and cancer risk in both sexes

	Men			Women		
	Cases*	Model 1^α^	Model 2^β^	Cases*	Model 1^α^	Model 2^β^
		HR (95%CI)	HR (95%CI)		HR (95%CI)	HR (95%CI)
All cancers						
Low	59,000	1.00	1.00	25,388	1.00	1.00
Moderate	12,676	**0.97 (0.95–0.98)**	0.99 (0.97–1.01)	4972	1.01 (0.98–1.04)	1.00 (0.97–1.03)
High-to-low	3659	0.98 (0.94–1.01)	0.99 (0.96–1.02)	1316	0.98 (0.93–1.04)	0.98 (0.93–1.04)
Low-to-high	3626	0.98 (0.95–1.02)	0.99 (0.96–1.03)	1342	0.98 (0.93–1.04)	0.97 (0.92–1.03)
High	3007	0.98 (0.95–1.02)	1.01 (0.98–1.05)	1093	0.94 (0.89–1.00)	**0.92 (0.87–0.98)**
Colorectum						
Low	8739	1.00	1.00	2669	1.00	1.00
Moderate	1799	**0.92 (0.88–0.97)**	0.95 (0.90–1.00)	479	0.97 (0.88–1.07)	0.96 (0.87–1.06)
High-to-low	524	0.95 (0.87–1.03)	0.97 (0.88–1.06)	149	0.94 (0.80–1.10)	0.93 (0.79–1.09)
Low-to-high	525	0.97 (0.89–1.06)	0.99 (0.91–1.09)	152	0.96 (0.82–1.13)	0.96 (0.82–1.13)
High	437	0.98 (0.89–1.08)	1.02 (0.93–1.13)	122	0.92 (0.77–1.10)	0.91 (0.76–1.10)
Liver						
Low	5512	1.00	1.00	837	1.00	1.00
Moderate	1173	0.96 (0.90–1.02)	1.00 (0.94–1.07)	158	1.03 (0.87–1.22)	1.05 (0.88–1.24)
High-to-low	321	0.97 (0.87–1.09)	1.00 (0.89–1.12)	67	1.25 (0.97–1.60)	1.24 (0.97–1.59)
Low-to-high	320	1.00 (0.89–1.12)	1.01 (0.90–1.13)	43	0.88 (0.66–1.19)	0.82 (0.60–1.11)
High	241	0.92 (0.81–1.05)	1.00 (0.88–1.14)	43	1.00 (0.74–1.35)	0.99 (0.73–1.35)
Lung						
Low	7069	1.00	1.00	1639	1.00	1.00
Moderate	1196	**0.76 (0.72–0.81)**	**0.88 (0.82–0.93)**	338	1.09 (0.97–1.22)	1.10 (0.97–1.23)
High-to-low	444	**0.89 (0.81–0.98)**	1.01 (0.91–1.11)	101	1.05 (0.86–1.28)	1.03 (0.84–1.26)
Low-to-high	474	0.93 (0.85–1.02)	1.01 (0.92–1.11)	99	1.02 (0.84–1.25)	1.03 (0.84–1.26)
High	309	**0.73 (0.65–0.82)**	0.90 (0.81–1.01)	89	1.07 (0.86–1.32)	1.08 (0.88–1.34)
Thyroid gland						
Low	3511	1.00	1.00	6461	1.00	1.00
Moderate	834	1.10 (1.02–1.18)	0.99 (0.92–1.07)	1339	1.05 (0.99–1.12)	1.03 (0.97–1.10)
High-to-low	152	0.89 (0.76–1.04)	**0.83 (0.71–0.98)**	277	0.99 (0.88–1.12)	0.99 (0.88–1.12)
Low-to-high	120	0.86 (0.72–1.03)	**0.80 (0.67–0.96)**	303	1.02 (0.91–1.14)	1.01 (0.90–1.13)
High	114	0.94 (0.78–1.13)	**0.82 (0.68–0.99)**	261	1.03 (0.91–1.16)	1.01 (0.89–1.14)
Breast						
Low				4444	1.00	1.00
Moderate				964	**1.11 (1.03–1.19)**	1.06 (0.99–1.14)
High-to-low				170	0.88 (0.76–1.02)	0.88 (0.75–1.02)
Low-to-high				182	0.87 (0.75–1.01)	0.88 (0.76–1.02)
High				150	0.86 (0.73–1.01)	**0.82 (0.70–0.96)**
Corpus uteri						
Low				573	1.00	1.00
Moderate				131	1.16 (0.96–1.40)	1.11 (0.92–1.35)
High-to-low				26	1.11 (0.76–1.62)	1.03 (0.70–1.53)
Low-to-high				21	0.81 (0.53–1.24)	0.78 (0.50–1.20)
High				28	1.21 (0.82–1.76)	1.18 (0.81–1.73)

* The number of observations was different between Model 1 and Model 2 due to the missing value of covariates. The number of cases was calculated for model 2

^α^ adjusted for age

^β^ adjusted for age, income, frequency of alcohol drinking, smoking status, BMI group, and Charlson Comorbidity Index

During the 4,615,930 person-years of follow-up, 35,049 women developed cancer. A negative association between moderate frequency of PA and breast cancer incidence was observed in the age-adjusted model; however, a significant association was not observed in the fully-adjusted model. After adjusting for covariates, a high frequency of PA during the 7 years was significantly associated with a decreased risk of all cancers (HR = 0.92, 95%CI = 0.87–0.98) and breast cancer (HR = 0.82, 95%CI = 0.70–0.96) (Table 3).

In the subgroup analysis by smoking status, in men, moderate frequency of PA was significantly associated with a lower risk for colorectal cancer in male smokers, while low-to-high and high frequencies of PA were significantly associated with a lower risk for thyroid cancer in non-smoking men. A significant association between moderate PA trajectory and lung cancer was observed in both non-smoking and smoking men. In women, a high PA trajectory was associated with a decreased risk for all cancers and breast cancer among non-smoking women, whereas a null association was observed in smoking women (Table 4).

**Table 4:** HRs and 95% CIs for the association between physical activity trajectory and cancer risk in both sexes, by smoking status

	Men		Women	
	Non-smoker	Smoker	Non-smoker	Smoker
	HR (95%CI)	HR (95%CI)	HR (95%CI)	HR (95%CI)
All cancers				
Low	1.00	1.00	1.00	1.00
Moderate	1.00 (0.97–1.02)	0.97 (0.95–1.01)	0.99 (0.96–1.03)	1.22 (0.96–1.55)
High-to-low	0.96 (0.92–1.00)	1.05 (0.99–1.10)	0.98 (0.93–1.04)	1.06 (0.72–1.54)
Low-to-high	1.00 (0.95–1.04)	0.99 (0.94–1.05)	0.97 (0.92–1.02)	1.20 (0.83–1.74)
High	1.01 (0.97–1.06)	1.03 (0.96–1.10)	**0.92 (0.87–0.98)**	0.95 (0.60–1.50)
Colorectum				
Low	1.00	1.00	1.00	1.00
Moderate	0.99 (0.93–1.06)	**0.89 (0.82–0.96)**	0.96 (0.87–1.06)	1.08 (0.53–2.19)
High-to-low	0.91 (0.81–1.02)	1.08 (0.94–1.25)	0.94 (0.79–1.11)	0.49 (0.12–2.01)
Low-to-high	1.00 (0.89–1.12)	0.99 (0.86–1.14)	0.97 (0.82–1.14)	0.56 (0.14–2.30)
High	1.03 (0.92–1.16)	1.02 (0.85–1.21)	0.92 (0.76–1.10)	0.76 (0.18–3.11)
Liver				
Low	1.00	1.00	1.00	1.00
Moderate	1.03 (0.94–1.11)	0.97 (0.88–1.07)	1.04 (0.87–1.24)	1.23 (0.42–3.59)
High-to-low	0.95 (0.82–1.11)	1.08 (0.91–1.28)	1.27 (0.99–1.63)	–
Low-to-high	1.02 (0.88–1.19)	0.99 (0.82–1.18)	0.80 (0.58–1.09)	1.43 (0.34–6.05)
High	0.91 (0.77–1.07)	1.19 (0.97–1.47)	1.02 (0.75–1.39)	–
Lung				
Low	1.00	1.00	1.00	1.00
Moderate	**0.88 (0.80–0.96)**	**0.88 (0.80–0.95)**	1.10 (0.98–1.24)	1.02 (0.49–2.15)
High-to-low	0.94 (0.81–1.09)	1.08 (0.95–1.23)	1.03 (0.84–1.26)	1.11 (0.45–2.78)
Low-to-high	1.02 (0.88–1.17)	1.02 (0.90–1.15)	1.00 (0.81–1.23)	2.01 (0.92–4.40)
High	0.89 (0.76–1.04)	0.94 (0.80–1.11)	1.09 (0.87–1.35)	1.19 (0.37–3.83)
Thyroid				
Low	1.00	1.00	1.00	1.00
Moderate	0.98 (0.89–1.08)	1.01 (0.88–1.15)	1.03 (0.97–1.09)	1.48 (0.86–2.55)
High-to-low	0.83 (0.69–1.01)	0.84 (0.62–1.13)	0.98 (0.87–1.11)	1.84 (0.79–4.26)
Low-to-high	**0.77 (0.62–0.97)**	0.87 (0.64–1.18)	1.00 (0.89–1.13)	1.43 (0.57–3.56)
High	**0.80 (0.64–1.00)**	0.88 (0.62–1.26)	1.01 (0.89–1.14)	0.97 (0.30–3.10)
Breast				
Low			1.00	1.00
Moderate			1.06 (0.99–1.14)	1.14 (0.59–2.19)
High-to-low			0.87 (0.74–1.02)	1.42 (0.51–3.93)
Low-to-high			0.87 (0.75–1.01)	1.79 (0.71–4.48)
High			**0.81 (0.69–0.95)**	1.42 (0.51–3.95)
Corpus uteri				
Low			1.00	1.00
Moderate			1.11 (0.91–1.34)	1.66 (0.34–8.11)
High-to-low			1.01 (0.68–1.51)	2.37 (0.29–19.46)
Low-to-high			0.75 (0.48–1.18)	2.45 (0.30–20.12)
High			1.21 (0.82–1.77)	–

Adjusted for age, income, frequency of alcohol drinking, smoking status, BMI group, and Charlson Comorbidity Index

In the subgroup analysis by BMI, a significant negative impact of moderate trajectory of PA on the risk for colorectal and lung cancers was observed in men who were underweight or had a normal BMI. There was an association between high-to-low PA trajectory and lower risk for thyroid cancer among overweight/obese men. Notably, the high PA trajectory was associated with an increased risk for corpus uteri cancer compared to the low PA trajectory (Supplemental Table 2).

## Discussion

Our study was the first to identify the trajectories of PA frequency and its relationship with all cancer risk and several specific cancers. Our findings showed that more than two-thirds of middle-aged Korean adults remained at a low frequency of PA, and only 5% had a high frequency of PA during approximately 7 years. Additionally, this study revealed that a small proportion of people changed their frequency of PA from low to high and from high to low, and each trajectory accounted for approximately only 5% in both sexes. Existing evidence suggests that PA has a protective effect on cancer prevention, and PA in almost studies was usually measured at a single time point (i.e., baseline) [19–23]. Hence, we hypothesized that PA trajectories during the 7 years could have modified the association between PA and cancer incidence. The present study unveiled a novel finding that, compared to persistent low frequency, maintaining a high frequency of PA over a period of approximately 7 years was significantly associated with a lower risk of all cancer incidence among women.

The link between PA and cancer risk, especially that of specific cancers, has been established in observational studies; however, to our knowledge, no study has assessed the effect of PA trajectories on the risk for all cancers. Limited research has shown an inverse impact of non-trajectory-based PA on the development of all cancers [24, 25]. In particular, a systematic review and meta-analysis of 47 studies involving 5,797,768 participants and 55,162 cases showed a decreased risk for digestive-system cancers in the high PA level group (Relative risk (RR) = 0.82, 95%CI = 0.79–0.85) compared to the low PA level group, and significant results were observed in both sexes [24]. A study pooling 1.44 million adults from 12 prospective US and European cohorts also indicated that a high level of baseline leisure-time PA was associated with lower risks for 13 cancers [25]. Additionally, the significant effect of high-to-low trajectory PA on cancer risk was not observed in our study, thereby emphasizing the importance of maintaining a high frequency of PA during a long period for the reduction of the risk for all cancers, rather than performing a high frequency of PA temporarily. However, the proportion of people with the persistent high trajectory of PA accounted for approximately 5%; therefore, promoting daily PA is crucial for preventing cancer development, especially in women. Although our study could not demonstrate the protective impact of the low-to-high trajectory of PA on cancer risk, we believed that if a high level of PA was maintained for a long duration, rather than persistently low PA, the risk for cancer development could be reduced. Therefore, further studies are required to assess PA trajectories over longer period to confirm its association with cancer risk. Our study also showed a favorable effect of PA on cancer prevention in women only after adjusting for potential covariates, such as smoking and alcohol consumption. In fact, men are more likely to use tobacco and drink alcohol than women, as also observed in our study, and both behaviors are the most important risk factors attributable to cancer development. Additionally, the higher cancer susceptibility among men due to exposure to carcinogens from work, unwillingness to seek healthcare or sex-related biologic factors could be latent factors [26], which could not be controlled in our study. Therefore, sex differences in lifestyle behaviors could explain the negligible effect of PA on all cancer risk in men observed in our study.

World Cancer Research Fund/American Institute for Cancer Research (WCRF/AICR) determined the relationship of total PA with lung cancer risk to be limited-suggestive evidence [2]. A recent meta-analysis of 20 cohort studies has demonstrated that increased PA was associated with a lower risk for lung cancer in both sexes and in smokers, but not in non-smokers [27], and this finding was consistent with other previous studies [28, 29]. In our study, we observed the pronounced association between lung cancer and a moderate level of PA in both smokers and non-smokers, but in men only. The heterogeneity among studies related to the link between lung cancer and PA level, including measurement of PA, study design, and study participants’ characteristics, is challenging, making the results of the numerous studies not comparable. Our study measured the frequency of general PA, while others focused on either recreational PA, non-recreational PA, or all domains of PA. The underlying mechanisms of PA can prevent lung cancer development through several pathways, including reducing insulin resistance and inflammation, decreasing oxidative stress and enhancing DNA repair mechanisms, increasing enzymatic systems and cofactors such as glutathione that detoxify chemical carcinogens, and enhancing the innate and acquired immune response [2]. Furthermore, the protective impact of PA on lung cancer prevention was seen in former smokers and non-smokers in our study. This could probably be attributed to the endogenous antioxidant defenses in the association between PA and lung cancer, as seen in the molecular epidemiology study within EPIC project [20]. Moreover, the relatively low number of lung cancer cases among women could reduce the statistical robustness that could interfere with the statistical significance observed in our study. Given that smoking is the most important factor attributable to lung cancer, prevention of smoking initiation and promotion of smoking cessation is the most effective method of primary prevention against lung cancer. Additionally, our study suggested that actively engaging in PA enormously contributes to lung cancer prevention in men, regardless of their smoking status.

The association between PA and thyroid cancer is inconclusive. The very first case-control study in the US established the hypothesis that regular recreational exercise reduced the risk for thyroid cancer (Odds ratio (OR) = 0.76, 95%CI = 0.59–0.98) and was also supported by other case-control studies in European countries [23]. Another case-control study in South Italy also suggested that walking every day for at least 60 min diminished the risk for thyroid cancer development (OR = 0.357, 95%CI = 0.157–0.673). However, evidence from large cohorts does not support this hypothesis. A study in the US involving 484,326 men and women revealed that the risk for thyroid cancer and its subtypes was unassociated with vigorous exercise [30]. Another large study pooling data from five prospective studies which measured different aspects of PA (i.e., frequency of vigorous activities, metabolic equivalent task, number of hours spent performing vigorous activities, or strenuous exercise) showed that all patterns of PA were insignificantly associated with thyroid cancer development [31]. A null association between leisure-time PA and thyroid cancer was also observed in a large cohort study involving 1.4 million adults from the US and European countries, and the relationship between PA and cancer risk was not modified by BMI or smoking status [25]. In contrast, our study emphasized that the risk for thyroid cancer was reduced among men who engaged in low-to-high, high-to-low, and persistently high trajectories of PA frequency in comparison with men who engaged in a persistently low trajectory of PA frequency. The inconsistent methods of measuring PA and its domains, volumes, and time periods across epidemiological studies make the results difficult to compare among studies. Sufficient evidence of a consistent method to determine the true association between PA and thyroid cancer is currently unavailable. Furthermore, evidence showed that the risk for thyroid cancer was strongly associated with obesity in men [32]; additionally, our study highlighted the effect of a higher frequency of PA in reducing thyroid cancer risk in overweight and obese individuals. Based on our study results and the fact that thyroid cancer is one of the five most common cancers in Korea [33], we highlighted the need for high frequency PA over a long period, especially among overweight and obese people.

The favorable impact of PA on breast cancer prevention was observed in postmenopausal women, and limited-suggestive evidence for this has been shown among premenopausal women by the WCRF/AICR [2]. A meta-analysis of 38 cohort studies investigated the inverse link of PA to breast cancer risk in both premenopausal and postmenopausal women, and this association was consistent among all domains of PA [34]. In line with previous studies, the reduction in breast cancer risk was observed in women who had a persistently high frequency of PA in our study, compared with those who had a low frequency of PA. This finding is similar to that reported in another large cohort study [25]. However, we could not examine the beneficial effects of PA against breast cancer in premenopausal and postmenopausal women separately due to the lack of such information; therefore, future research is required to investigate the confounding effects of women-health variables, including menopause, parity, and hormone replacement therapy, on the relationship between trajectories of PA and breast cancer risk. Regular PA via a diverse array of mechanisms, such as reduction in circulating estrogens levels, insulin resistance, and inflammation, was reported to have a protective impact against breast cancer [2]. We could not observe the significant link between PA trajectory and breast cancer risk in the subgroup analyses by BMI. This could be due to the relatively small sub-population.

Convincing evidence demonstrated that the highest level of total PA reduced the risk for colon cancer by 20% versus the lowest level (RR = 0.80, 95%CI = 0.72–0.88) [2]; in contrast, PA had a negligible effect on the prevention of colorectal cancer in our study. A significant inverse association between PA and colorectal cancer in men did not remain after adjusting for potential confounders, such as alcohol consumption, smoking, BMI, and CCI. As for women, a null association between PA and colorectal cancer was seen in the present study, consistent with 13 other studies with RRs ranging from 0.69 to 1.15, whereas another study showed an increase of PA between baseline and follow-up showed the beneficial effect of PA on the prevention of colon cancer only, not of rectal cancer and combined colorectal cancer [35]. In the subgroup analysis, we observed a decrease in the risk for colorectal cancer in male smokers who maintained a moderate level of PA, similar to another large cohort study that measured the effect of PA on risk for colon and rectal cancer independently [25]. Obesity is another established risk factor for colorectal cancer, especially in men [36]. In our study, moderate PA was insufficient to lower the risk for colorectal cancer in overweight/obese men, even though significant results were seen in underweight and normal men. Based on this finding, we placed a strong emphasis on weight management in conjunction with PA to maximize cancer risk prevention.

Findings regarding the impact of PA on endometrial cancer risk in women have been largely equivocal as some studies showed an inverse association [37, 38], while others reported no link [39], similar to the findings of our study. However, it is notable that a higher risk for corpus uteri cancer was associated with the persistently high frequency PA group in underweight and normal BMI persons. To the best of our knowledge, this finding has not been previously reported in the literature. In contrast, other studies indicated that high lifetime PA was linked to a higher risk for endometrial cancer in overweight and obese women [38]. We hypothesized that a lack of women-health covariates in our study could modify the effect of PA on cancer risk. In addition, given the limited sample size in sub-categories, larger studies that are adjusted for sufficient covariates will be needed to clarify the role of BMI in the relationship between PA and corpus uteri cancer before firm conclusions can be drawn.

Additionally, while our study observed a null association between PA trajectory and risk for liver cancer, WCRF/AICR showed evidence that the association between liver cancer and PA was “limited-suggestive” [25], and a meta-analysis demonstrated an inverse relationship between daily total PA and liver cancer risk [40]. The discrepancies in the measurement of various PA domains and time periods could confound the relationship of PA with the risk for these cancers. Therefore, a sufficient number of studies with homogeneous methods of PA assessment are needed to confirm the true association of PA with the risk for liver cancer.

Our study had several limitations. First, since the NHIS cohort did not use the global PA questionnaire developed by the WHO [41], we could not collect detailed information regarding certain domains of PA and did not measure the metabolic equivalents of the tasks. For example, people who did PA regularly between 60 and 70% of their target heart rate may report that they did not sweat during, which may underestimate the effect of PA on cancer prevention. Second, Due to only including participants who received health examinations, this cohort study was unable to represent the entire Korean population. Furthermore, the fact that there were twice as many men as women can be attributed to the fact that most of the men were employed and the head of family, who were eligible for the general health screening program. Meanwhile, only individuals aged 40 years and older who were the dependents of the employed person and head of household were eligible for the program, and the majority of them were women. It also contributed to the fact that women were older than men in our study [10]. Third, the cancer incidence in this study was defined based on ICD-10 codes in primary diagnosis and a special code for verifying cancer in the claims data; however, a secondary diagnosis of cancer was not considered. This could lead to an underestimation of cancer incidence. Forth, despite the enormous number of participants, the small number of incident cases of rare cancer in the trajectory strata could reduce the statistical power. Finally, the study lacked an additional set of covariates significant in developing specific cancer sites (e.g., menopause status in relation to breast cancer), which could potentially confound the association of PA with cancer risk. Therefore, cancer-specific covariates should be considered as confounding factors in further analyses.

## Conclusions

More than two-thirds of the middle-aged Korean population had a low frequency of PA for approximately 7 years. Compared to persistent low frequency, maintaining a high frequency of PA was significantly associated with a lower risk for the onset of all cancers and breast cancer in women; and thyroid cancer among men. A reduction in the risk for lung and colorectal cancer was also observed for smoking men who had a moderate level of PA frequency relative to those who had a low level of PA frequency. Thus, our study suggests that increasing physical activity as part of the daily routine should be widely promoted to protect individuals against cancer development for women.

## Supplementary Information

**Additional file 1.**

## Data Availability

The datasets generated and analyzed in the current study are available upon request from the National Health Insurance Sharing Service (https://nhiss.nhis.or.kr/).

## Abbreviations

HR: Hazard ratio
CI: Confidence interval
PA: Physical activity
NHIS: National Health Insurance Services
GBTM: Group-based trajectory modeling
ICD-10: International Classification of Diseases 10th edition
BIC: Bayesian Information Criteria
BMI: Body mass index
CCI: Charlson Comorbidity Index
RR: Relative risk
WCRF/AICR: World Cancer Research Fund/American Institute for Cancer Research
OR: Odds ratio
