# Supplementary material for: Trajectory of physical activity frequency and cancer risk: Findings from a population-based cohort study
Source: Eur Rev Aging Phys Act. 2023 Mar 9;20:4. doi: 10.1186/s11556-023-00316-5 (PMC9996897; doi:10.1186/s11556-023-00316-5)
Supplement: Supplementary file 1 — Additional file 1. [file 11556_2023_316_MOESM1_ESM.docx]

**Supplemental Table 1: Comparison of BIC between the selected fitted model according to number of groups, by sex**

| **Number of groups** | **Polynomial** | **BIC** | **Group membership** |
| --- | --- | --- | --- |
| Men |  |  |  |
| 2 | 21 | -5827761 | 85.7%; 14.3% |
| 3 | 112 | -5756517 | 15.9%; 79.7%; 4.4% |
| 4 | 1111 | -5694271 | 6.4%; 15.2%; 75.7%; 2.8% |
| **5** | **01221** | **-5607559** | **73.5%; 3.9%; 16.2%; 3.48%; 2.9%** |
| 6 | 121221 | -5535186 | 2.0%; 3.0%; 70,4%; 3.4%; 19.1%; 2.3% |
| Women |  |  |  |
| 2 | 12 | -2922902 | 85.9%; 14.1% |
| 3 | 211 | -2877002 | 10.6%; 81.6%; 7.8% |
| 4 | 2122 | -2821515 | 4.3%; 75.5%; 16.3%; 3.0% |
| **5** | **11221** | **-2789923** | **14.6%; 74.7%; 3.7%; 3.8%; 3.3%** |
| 6 | 111121 | -2793302 | 73.9%; 2.0%; 11.4%; 7.4%; 3.3%; 2.0% |

BIC, Bayesian Information Criteria.

**Supplemental Table 2: HRs and 95% CIs for the association between physical activity trajectory and cancer risk in both sexes, by BMI group**

|  | **Men** | | **Women** | |
| --- | --- | --- | --- | --- |
|  | Underweight/Normal | Overweight/Obesity | Underweight/Normal | Overweight/Obesity |
|  | HR (95%CI) | HR (95%CI) | HR (95%CI) | HR (95%CI) |
| All cancers |  |  |  |  |
| Low | 1.00 | 1.00 | 1.00 | 1.00 |
| Moderate | 0.97 (0.93-1.00) | 1.00 (0.98-1.02) | 1.01 (0.96-1.05) | 0.99 (0.96-1.03) |
| High-to-low | 1.02 (0.96-1.08) | 0.98 (0.94-1.02) | 0.90 (0.82-1.00) | 1.02 (0.96-1.09) |
| Low-to-high | 1.02 (0.96-1.08) | 0.98 (0.94-1.03) | 0.92 (0.83-1.01) | 1.00 (0.93-1.07) |
| High | 1.07 (1.00-1.14) | 1.00 (0.95-1.04) | 0.91 (0.82-1.01) | 0.93 (0.86-1.00) |
| Colorectum |  |  |  |  |
| Low | 1.00 | 1.00 | 1.00 | 1.00 |
| Moderate | **0.87 (0.79-0.97)** | 0.98 (0.92-1.04) | 1.00 (0.85-1.17) | 0.94 (0.83-1.06) |
| High-to-low | 1.12 (0.96-1.31) | 0.91 (0.81-1.01) | 1.08 (0.82-1.41) | 0.86 (0.69-1.06) |
| Low-to-high | 0.94 (0.79-1.11) | 1.02 (0.92-1.13) | 0.82 (0.60-1.12) | 1.03 (0.85-1.25) |
| High | 1.16 (0.97-1.39) | 0.98 (0.87-1.10) | 0.82 (0.59-1.15) | 0.96 (0.77-1.19) |
| Liver |  |  |  |  |
| Low | 1.00 | 1.00 | 1.00 | 1.00 |
| Moderate | 0.97 (0.86-1.09) | 1.02 (0.95-1.10) | 1.11 (0.83-1.50) | 1.01 (0.82-1.24) |
| High-to-low | 1.09 (0.90-1.33) | 0.96 (0.84-1.10) | 0.99 (0.59-1.66) | 1.34 (1.00-1.78) |
| Low-to-high | 0.96 (0.78-1.18) | 1.04 (0.91-1.19) | 0.76 (0.42-1.39) | 0.84 (0.59-1.20) |
| High | 1.14 (0.90-1.43) | 0.95 (0.81-1.11) | 0.89 (0.47-1.67) | 1.02 (0.72-1.46) |
| Lung |  |  |  |  |
| Low | 1.00 | 1.00 | 1.00 | 1.00 |
| Moderate | **0.76 (0.68-0.85)** | 0.94 (0.87-1.02) | 1.08 (0.89-1.29) | 1.11 (0.95-1.30) |
| High-to-low | 0.93 (0.79-1.09) | 1.05 (0.93-1.19) | 0.85 (0.59-1.21) | 1.14 (0.89-1.46) |
| Low-to-high | 0.98 (0.85-1.14) | 1.03 (0.91-1.17) | 0.94 (0.66-1.33) | 1.08 (0.85-1.39) |
| High | 0.85 (0.70-1.04) | 0.93 (0.81-1.07) | 1.31 (0.95-1.80) | 0.96 (0.72-1.28) |
| Thyroid |  |  |  |  |
| Low | 1.00 | 1.00 | 1.00 | 1.00 |
| Moderate | 0.92 (0.78-1.09) | 1.01 (0.93-1.10) | 1.05 (0.96-1.15) | 1.02 (0.94-1.10) |
| High-to-low | 0.94 (0.68-1.30) | **0.81 (0.67-0.97)** | 0.89 (0.72-1.10) | 1.06 (0.91-1.22) |
| Low-to-high | 0.77 (0.52-1.14) | 0.82 (0.67-1.01) | 0.97 (0.80-1.19) | 1.03 (0.90-1.19) |
| High | 1.19 (0.84-1.69) | **0.73 (0.58-0.91)** | 0.88 (0.71-1.09) | 1.10 (0.94-1.28) |
| Breast |  |  |  |  |
| Low |  |  | 1.00 | 1.00 |
| Moderate |  |  | 1.06 (0.95-1.17) | 1.07 (0.97-1.18) |
| High-to-low |  |  | 0.86 (0.67-1.10) | 0.89 (0.73-1.08) |
| Low-to-high |  |  | 0.91 (0.71-1.16) | 0.87 (0.72-1.05) |
| High |  |  | 0.83 (0.64-1.07) | 0.81 (0.66-1.00) |
| Corpus uteri |  |  |  |  |
| Low |  |  | 1.00 | 1.00 |
| Moderate |  |  | 1.21 (0.89-1.64) | 1.06 (0.83-1.36) |
| High-to-low |  |  | 1.02 (0.50-2.07) | 1.03 (0.64-1.66) |
| Low-to-high |  |  | 0.62 (0.26-1.51) | 0.84 (0.51-1.39) |
| High |  |  | **1.85 (1.07-3.18)** | 0.87 (0.51-1.48) |
